# Light-curing dental resin-based composites: How it works and how you can make it work

**DOI:** 10.3389/fdmed.2023.1108316

**Published:** 2023-03-07

**Authors:** David C. Watts

**Affiliations:** School of Medical Sciences and Photon Science Institute, University of Manchester, Manchester, United Kingdom

**Keywords:** bulk fill, curing protocol, irradiance, light-curing units, photoinitiator, photo-polymerisation, reciprocity hypothesis, resin-composite

## Abstract

**Aim:**

Clinicians may become quite familiar with the rapid transformation of composite pastes to rigid solids as a routine phenomenon in operative dentistry. But they may still lack scientific understanding of how and why this happens. Efforts to learn scientifically about the interaction between light beams and resin-composites can significantly promote effective clinical placement of restorations. Neglect of such study can result in practical procedures of light curing that are inadequate or even seriously defective.

**Method:**

This review addresses the underlying science and technology to elucidate how light curing works for dental resin-based composites, including—but not limited to—bulk fill types. This involves questions concerning: (a) the particle-wave understanding of light; (b) how photons can penetrate sufficiently deeply into bulk fill composites; (c) the necessary technology of LED light-curing units (LCUs); (d) the criteria for absorption of photons by photoinitiators to initiate free-radical addition polymerisation.

**Conclusions:**

The implications for clinical practice are surveyed. These include design variables and selection criteria for LED-LCUs and guidelines on their use. This is to guide practitioners towards safe and effective light-curing procedures so that they can achieve optimal result for their patients.

## 1. Introduction

Resin-based composites (RBCs) are the most prevalent class of material for the direct restoration of teeth. They are commonly supplied by manufacturers as single pastes—within syringes or as a single dose “compule”—for extrusion and placement into a prepared dental cavity. Such clinical preparation normally requires prior application of an adhesive layer to the cavity floor and walls, as most RBCs are not self-adhesive to enamel or dentine. Composites are hardened *in situ* by the process and procedures of photo-polymerisation or photo-curing. This requires application of visible blue light from a suitable light-curing unit (LCU).

Over the past 15 years, manufacturers have developed RBC formulations capable of photo-polymerisation in a single increment of 4 mm thickness (or depth) by irradiation from an occlusal surface. These RBCs, exhibiting a Depth of Cure (DoC) of 4 mm or greater, are known as bulk-fill (BF) composites. Prior to their development typical DoCs were about 2 mm, requiring several layers or increments to fill a deep cavity.

The objective of this review is to address two main questions: “How does light curing work?” and “How can the clinician make it work?” This requires a brief review of the science of light itself, the current technology of light-curing units, based on light-emitting diodes (LEDs), and the modes of interaction of light with substances such as composites—especially their incorporated photoinitiator (PI) molecules. The procedural aspects address the practical issues that arise in the dental clinic and an appreciation of the several adverse consequences of under-curing resin-composites. The important prior issues of adhesive formulation, selection and application are not part of our present focus.

## 2. The nature of light

What is light? We are aware that light beams “travel in straight lines” but can change direction as seen in the phenomenon of refraction. White light can be split into its constituent colours by a prism or by atmospheric water droplets, producing rainbows. As to its intrinsic nature, over the past three centuries, *particle* and *wave* models have competed for dominance. However, thanks to quantum theory, a truce has been declared. Paradoxically, both models are now considered to be “true”. Light behaves as a stream of particles (*photons*), but collectively—or even single photons—exhibit wavelike characteristics, including interference and diffraction (1, 2). The photon (particle) concept is essential to explaining the photoelectric effect, the mechanism behind the operation of solar panels and digital cameras.

In terms of waves, a light beam has a *wavelength* (*λ*): the distance between successive peaks or troughs. This can be re-expressed as a *frequency* (*ν*), reciprocally related *via* a simple equation involving the *velocity* (c) of light in a vacuum.


(1) c=ν×λ

**Figure 1:**
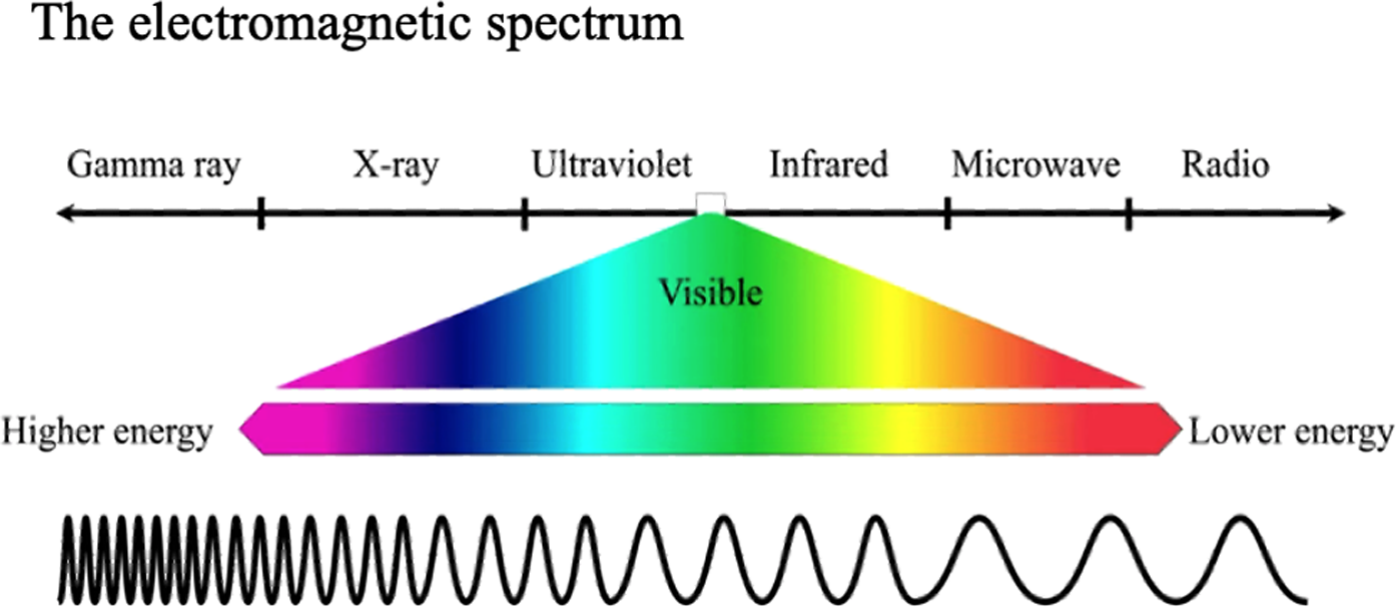
The central part of the electromagnetic spectrum consists of light of wavelengths visible to human eyes, ranging from violet to red. When seen together they are apparent as “white” light.

A beam of visible (white) light consists of a range (or spectrum) of wavelengths (or frequencies). As Isaac Newton showed, white light can be split *via* a glass prism into its constituent wavelength ranges, *from red to violet*, often denoted by the capital letters: ROYGBIV. As James Clark Maxwell showed, theoretically, and Heinrich Hertz showed experimentally, visible light is merely a central part of the whole electromagnetic spectrum (Figure 1) with *ultra-violet* (UV) extending beyond the violet and *infra-red* (IR) and then radio waves extending beyond the red.

According to quantum theory, each photon of light has an energy (E) given by the product of its frequency (*ν*) and Max Planck's constant (h).


(2) E=h×ν=h×c/λ

Planck's constant is almost unimaginably small (6.62607004 × 10^−34^ m^2^ kg/s). So, contrary to popular parlance, a *quantum leap* is the *smallest* possible change in energy! This also means that a solitary blue photon has only a small quantity of energy.

## 3. Units for expressing the quantity of light from a source

In everyday speech, the light delivered from any source is commonly referred to as its brightness or intensity. However, when the light output is quantified by a suitable radiometric instrument it is important to understand and use the correct technical terms and units.

In the International System of Units (SI), the **watt** (symbol: W) is a unit of **power** or radiant flux expressing the *rate* of **energy** transfer. Thus ***Power*** (W) is defined as ***energy*** (Joules) *per unit time*, or specifically: **Joules per second**.

As light emerges from the LCU light guide or “optic” over a defined exit area, this is expressed as the ***radiant exitance*** (UNITS **W/m^2^** or mW/cm^2^). Thus, when using a Light-Curing Unit (LCU), the radiant exitance (mW/cm^2^) is a measure of output ***power*** (Watts) *per unit area*.

Light that emerges from an LCU light guide is then intended to fall on the “target” surface. When light falls—or is incident—on a surface, the amount of light *received* is termed the **Irradiance** because—if the light beam from the optic tip is divergent—then the “concentration” of the beam will diminish with distance from the tip. Thus, considering the light energy falling on a target surface, we use the term Irradiance (**I**), but with the same units as radiant exitance (also mW/cm^2^), to allow for the fact that numerically, irradiance may be less than radiant exitance.

If, *at a fixed distance between tip and target surface*, the Irradiance remains constant *over time* (**t**), the **radiant exposure** or energy (E) delivered *per unit area* is:

$$\mathrm{Radiant}\;\mathrm{Exposure}=\mathrm{Energy},\;\mathrm{per}\;\mathrm{unit}\;\mathrm{area}\;\left(J/cm^{2}\right)=\mathrm{Irradiance}\;\left(W/cm^{2}\right)\times \mathrm{Time}\;\left(s\right)$$

or

(3) E=I×t

Alternatively, we can write:

TotalEnergyoutput(J)=TotalPoweroutput(W)×Time(s)

or

(4) E=P×t

The above equations are the main ones for understanding this subject. But understanding involves thinking about their physical meaning, their units and magnitudes, and how they interrelate.

A given LCU may be operated in the same output mode but with optic tips of different diameter. In either case, its Total Power output (W) will remain constant (Equation 4). But if used with a tip of smaller diameter, the Irradiance (W/cm^2^) will be greater than with a tip of larger diameter (Equation 3). Comparison of LCUs by their Total Power output avoids the area-dependent ambiguities of Radiant Exitance or Irradiance. Nevertheless, the measurement of irradiance is more meaningful for dental application given the importance of spatial control for effective material polymerisation.

When we use a torch or a light-curing unit, it is conceptually helpful to think of this as emitting a continuous stream of photons. Even LCUs that deliver a relatively modest irradiance, emit some *billion billion* (10^18^) photons every second. However, these photons are not all necessarily “suitable”. Their suitability depends upon their frequency or spectral wavelength. Most LED-LCUs output visible blue light of a wavelength of *circa* 470 nm. But solid-state “chips” emitting shorter wavelength violet light, *circa* 410 nm, may also be used, as discussed further in section 6.2.

Before considering what happens to these photons, we must briefly review the composition of RBCs—that also applies to bulk fill formulations—and the technology of LCUs based on light-emitting diodes (LEDs).

## 4. Formulation of resin-based composites

All RBCs are formulated with monomer (resin) mixtures that can be polymerized to form a solid organic resin matrix. Monomers in current formulations are predominantly *dimethacrylates* that incorporate pairs of carbon-carbon *double* bonds (C = C): one at either end of each monomer molecule. Different types of organic structures can exist between the C = C groups that vary in stiffness/flexibility and length (or size). Examples are the well-known structures of bis-GMA, UDMA and TEGDMA. It is the C = C bonds that undergo polymerization to create *single* C-C bonds in their place, linking the original monomers into a 3D polymer network structure, with extensive cross-linking, rather than either linear or branched polymer chains (3). Network structure formation causes a rapid increase in elastic modulus (i.e., stiffness, per unit cross-section) and increases local molecular density (3, 4), that corresponds to bulk polymerization shrinkage (5, 6).

**Figure 2:**
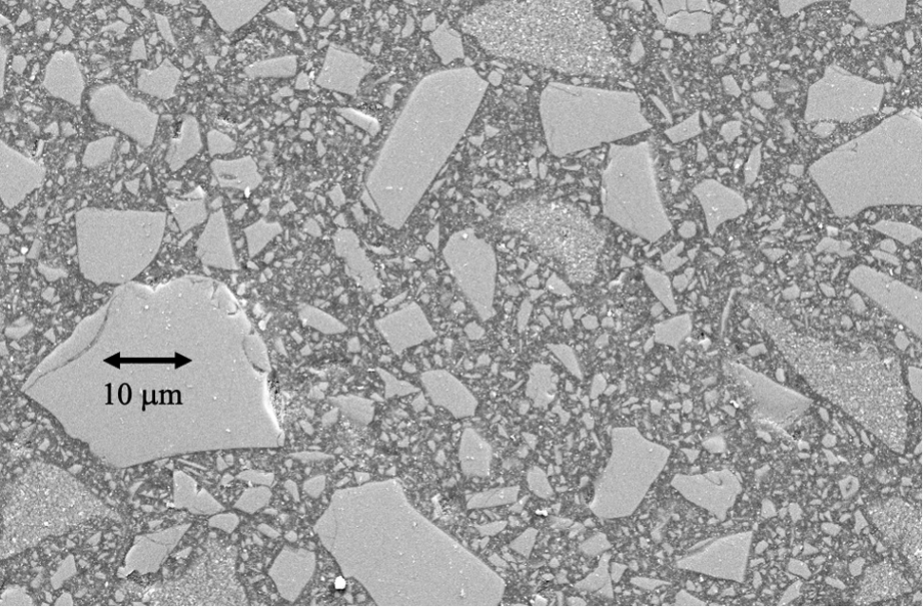
Scanning electron microscope image of a representative contemporary resin-based BF composite. The larger incorporated particles (>10 µm) are themselves fragmented chips of pre-polymerised composite, rather than solid inorganic powder particles. These pre-polymerised particles allow for finishing and polishing after placement to a high gloss.

Pre-dispersed within the monomers are high volume fractions of inorganic filler particles (7) frequently including pre-polymerized composite fragments (8) (Figure 2). These (mainly inert) particles are normally coated with a silane coupling agent that can co-polymerize with the resin matrix (9, 10). These components are designed to create strong, stiff restorative materials that bear some comparison, both structurally and in properties, to the major tissues (enamel and particularly dentine) that the RBC is intended to repair. This outcome depends upon successful photopolymerization of the resin phase. To achieve this goal, photoinitiator (PI) system molecules are also pre-dispersed within the resin-phase at a concentration of *circa* 0.1–0.2 percent, depending on whether this is expressed as a mass or mol%.

## 5. How do light-curing units work?

Quartz-tungsten halogen (QTH) LCUs were previously widely used in dentistry. These transformed restorative dentistry but had several disadvantages, which were definitively overcome by blue light-emitting diode (LED) LCUs.

LEDs are generically solid-state devices that emit visible radiation when an electrical potential (voltage) is supplied to suitable materials. This electroluminescence was discovered by accident early in the last century and the first LED results were published in 1907 (11). LEDs were forgotten only to be rediscovered in the 1920s and again in the 1950s. The first viable LEDs were by-products of research into semiconductor lasers. During the past 60 years, LEDs have become devices in their own right and today are versatile light sources, with extensive domestic and industrial applications. State-of-the-art LEDs are small, rugged, reliable, bright and reasonably energy-efficient (12). The 2014 Nobel Prize in Physics was awarded to three Japanese scientists for the invention of blue LEDs (13).

The possible dental application of blue LEDs was first proposed by Mills in 1995 (14) and shown to be viable by Mills, Jandt and Ashworth in 1999 (15). At that time, the radiant emittance of available LEDs was relatively low, so concentric arrays of LED chips were designed and patented (16) and the first dental LED-LCU publications appeared in 1999 (15, 17–20)]. Production of commercial LED-LCU devices for dentistry was facilitated by the development of high irradiance single *LumiLed* chips (20, 21).

Although LED devices are the most energy-efficient light sources we have, (about 30%–40% efficient, compared with 1% for incandescent bulbs) LED chips still produce significant quantities of heat from the supplied electrical power. Heat energy from LCUs is transferred to the optic tip mainly by non-radiative means such as conduction. This contributes to transient temperature increases (22–25), depending upon the LED design and exposure duration, as discussed below (section 7.4).

### 5.1. Beam profiles

**Figure 3:**
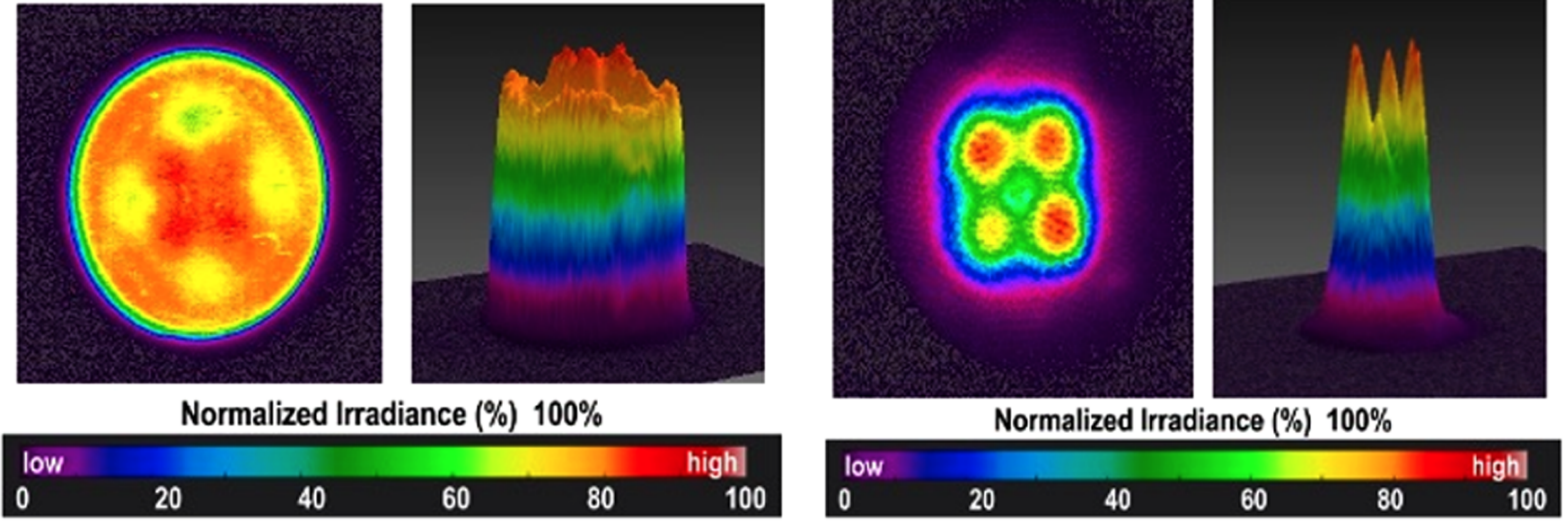
Beam profiles measured for well-designed LED-LCUs. Both these units emit light from 2 × 2 arrays of solid-state chips.

When photons are emitted from the light guide of a LED-LCU it is tempting to assume that they are of *uniform and constant number density* across the surface of the optic tip. An underappreciated feature of LED LCUs is that the light output across the optic tip area can vary considerably. But even if it were safe to visually assess the beam profile, it would be impossible to differentiate irradiance variations across the LCU tip. However, this can be measured by a high-resolution digital camera with a ground-glass plate placed in front of the optic tip. Usually, an optical bench setup is necessary to ensure alignment and measurement reproducibility. Figure 3 shows representative profiles from two well-designed LCUs, exhibiting high levels of uniformity across the beam. Unfortunately, many inferior designs are available that have dangerous “hot” and “cold” spots within the beam.

**Figure 4:**
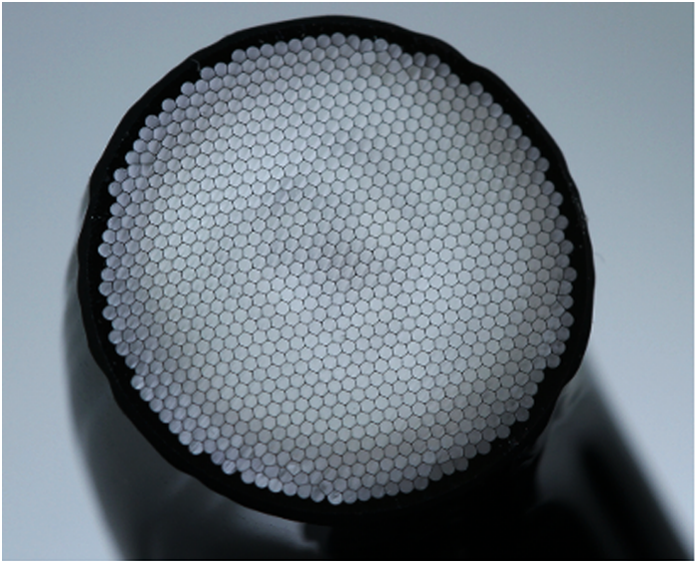
Construction of a LCU optic tip containing a glass-fibre bundle (Bluephase PowerCure, Ivoclar AG).

The construction of a typical optic tip is shown in Figure 4, consisting of an array of glass fibres surrounded by “cladding”.

## 6. How photons interact with resin-composites

There are two main questions:

1. How deeply do these photons penetrate bulk-fill RBCs?
2. What happens when a suitable photon meets a photo-sensitive molecule within the resin part of the resin-composite.

**Figure 5:**
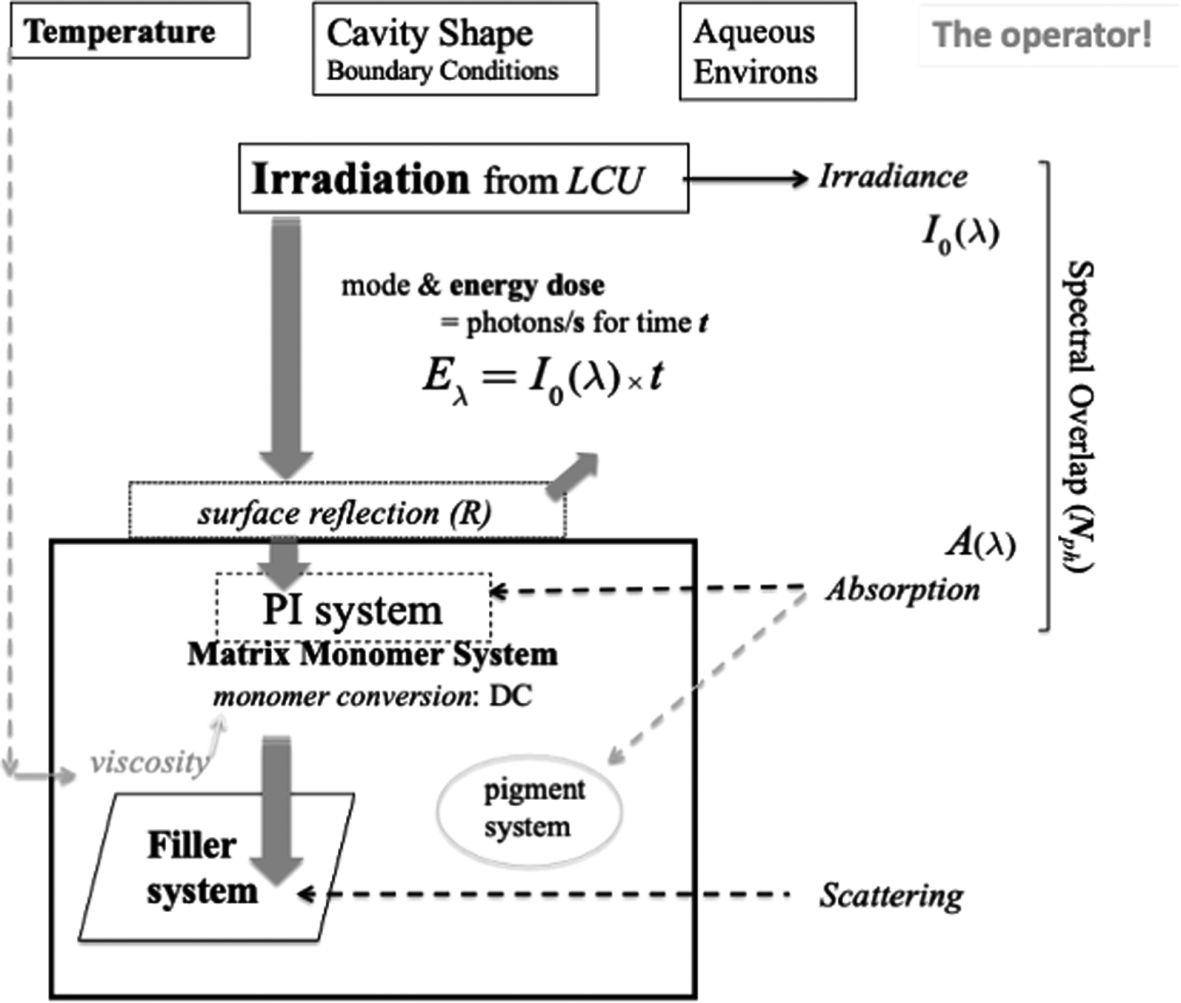
Schematic of irradiation and photo-initiation of a resin-composite system. The penetration and interaction of visible photons with the composite formulation is represented in the context of the extrinsic factors of the dental-clinical environment and boundary conditions. The clinical operator also plays a crucial role in managing the process. Light scattering arises principally at the interfaces of reinforcing particles. However, any resin-phase porosity—if present—can also induce scattering.

Figure 5 shows formally what happens during dental light curing, referring to several intrinsic and extrinsic variables. These will be considered and explained sequentially.

### 6.1. Light penetration into resin-composite restoratives

**Figure 6:**
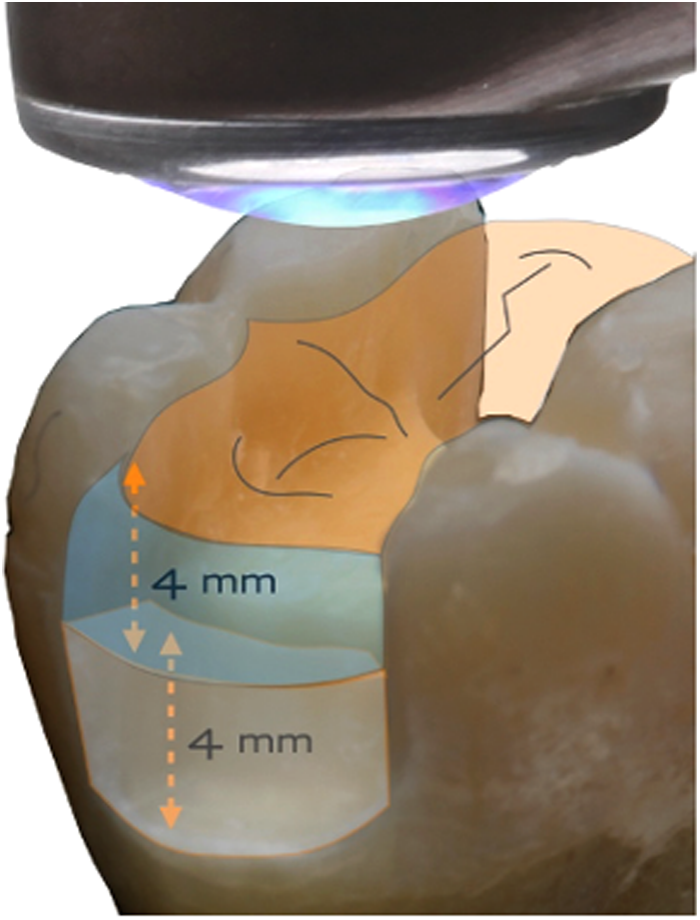
Even with direct contact of the light guide tip and the occlusal surface, there can be a finite distance to the proximal box.

Firstly, as the stream of photons from the LCU optic tip reaches the surface of the target composite, some may be lost if the optic tip is any distance from the target. This is due to the divergence angle of the light beam, whereby the irradiance generally decreases with distance from the tip (26–30). That is why the distinction between *radiant emittance* and *irradiance* is important. These quantities are only numerically equal when the tip is in immediate proximity to the target. Clinically, this is not always possible; for example, in a Class I or Class II cavity, the remaining cusps may create a “standoff” for the optic tip, above the occlusal surface of the restoration, (Figure 6).

Secondly, when light is incident on the RBC-paste surface a significant fraction may be *reflected* back, as expressed by the quantity **r** in Equation 5.

Thirdly, light that penetrates within the top surface of the composite will be subject to *attenuation via* two main processes: (i) absorption and (ii) scattering. The combined effect of these processes is characterised by the Beer-Lambert law (31), that expresses an exponential *decrease* of irradiance (***I***) with depth (**d**), with an *attenuation coefficient* (*μ*).

(5) $$I=I_{0}(1-r)e^{-\mu \;d}$$

where ***I*_0_** is the irradiance incident upon the top surface and **r** is the fraction of light undergoing specular and/or diffuse reflection from the surface.

(6) $$\mu =\mu_{a}+\mu_{s}$$

The (Naperian) attenuation coefficient (*μ*) is the sum of the coefficients for absorption and scattering, as per Equation 6,

The absorption coefficient (*μ_a_*) is related to an important quantity: the molar absorptivity (*ε*) (or extinction coefficient) and the concentration (C) of the absorbing molecule. This is essentially a statement of Beer's law (31):

(7) $${\;\mu }_{\;a}=\;\varepsilon \times \;C$$

Scattering of light is commonplace at internal interfaces, especially where there is a difference of refractive index (**n**) between two phases, such as resin and filler particles (32). Scattering increases appreciably with shorter wavelengths, so blue light penetrates more than violet light (33, 34). Filler-particle size has a major effect (32, 35, 36). When particle or fibre diameters are *greater* than the wavelength of light (*ca.* 470 nm or 0.47 µm), the light beam “sees” the particles and is refracted as it passes through, *i.e.,* scattered from its original direction of travel (35, 36). By contrast, nanoparticles (*ca.* 100 nm) are not “seen” by the light beam and so do not scatter light.

The art and science of RBC formulation takes these physical factors into account to mitigate undesired effects. This has been particularly critical in designing bulk fill materials with optimized light transmission and using high-efficiency photoinitiator mixtures; (see 6.4).

Absorption of light occurs as photons encounter: (a) pigment molecules or similar species and (b) photoinitiator (PI) molecules. We will now consider PI systems in more detail.

### 6.2. Photons encounter photoinitiators

Photosensitive compounds occur rather widely in the natural world. The best-known example is *chlorophyll* in plants and cyanobacteria; its colour is green because it mainly absorbs blue and red wavelengths from sunlight.

**Figure 7:**
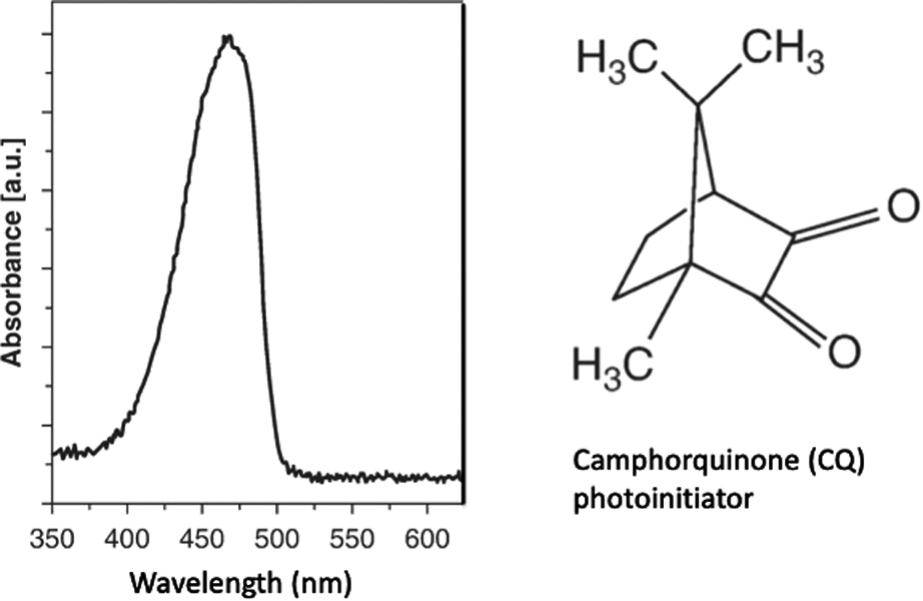
Camphorquinone (CQ) photoinitiator absorbs light in the blue region of the visible spectrum.

Light-curing units (LCUs) for dentistry normally must deliver light from the blue region of the visible spectrum that corresponds to the wavelength range over which photoinitiator(s) incorporated in resin-monomers can absorb energy. The first visible-light photoinitiator system for dentistry was developed and patented in 1975 at the Corporate Laboratories of Imperial Chemical Industries (ICI) PLC in the United Kingdom (37). This used Camphorquinone (CQ) as the photo absorber (Figure 7) in combination with an amine. CQ is a yellow compound, as it absorbs blue wavelengths (*ca.* 470 nm) from visible light. Within dental RBCs, *suitable* photoinitiator systems respond to (absorb) visible blue and/or violet light.

**Figure 8:**
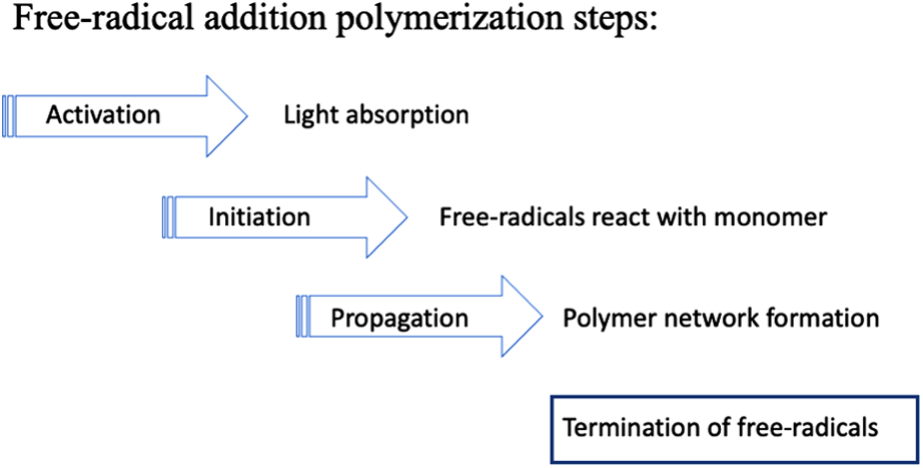
Free-radical polymerization involves successive steps. *Initiation* creates monomer molecules with unpaired electrons. During *propagation* these radicals combine with further monomers forming growing polymer chains. Eventually the growth process stops due to one or more *termination* reactions.

PI systems may be classified into two types: Norrish Type I and Norrish Type II. Camphorquinone/amine is a Type II system. The absorption of photons transforms CQ molecules to an excited (higher energy) state that is then capable of reaction with an amine co-initiator to generate free radicals. More recently, Type I systems have also been used that involve a simpler bond-cleavage mechanism. Both types result in the formation of free radicals, i.e., highly reactive molecules with an unpaired electron. These start a photochemical process that initiates *free-radical addition polymerization* reactions. The propagation of the polymerization reaction involves radical-ended chains reacting with successive monomer molecules (Figure 8).

Some PIs absorb more strongly in the shorter-wavelength violet region (*ca.* 410 nm) as compared to the blue region (*ca*. 470 m). Alternative PIs may also avoid any residual yellow coloration in the composite, post-irradiation. This is particularly important for design of white shades of resin-composites suitable for patients after tooth-whitening.

This availability of alternative photoinitiators has promoted the design and the commercial production of LED-LCUs incorporating two types of LED chip—of either *ca*. 470 nm (blue) or *ca*. 410 nm (violet) peak-spectral output (38). Some devices have one *ca*. 410 nm chip and three *ca*. 470 nm chips in a 2 × 2 array (Figure 3). Accordingly, blue and violet photons are emitted together and emerge *via* the LCU-optic (light guide). Sometimes, such devices are named “polywave” although that term may be commercially copyright. “Multi-chip” is an acceptable alternative. Again, this type of output has been termed wide-band or broad-band. But, if these terms are used, it must be understood that the emitted spectrum may consist of overlapping spectral peaks. Violet light—being of shorter wavelength than blue light—has reduced penetrative effectiveness through resin-based composites (RBCs), due to greater scattering and absorption effects. This may be significant when photo-curing bulk fill materials to 4 mm or greater depths.

Suitability, of a PI system, means that it corresponds to or matches the output wavelengths of the light-curing unit by having an absorption band within the output wavelength range (23, 24). This is illustrated schematically in Figure 5. Comparison might be made with a successful postal delivery. It is not sufficient to take a letter or parcel to a destination; there must be a letter box large enough to receive the letter (unless the door is opened)! So, the critical light energy “delivered” is that which *reaches* its intended destination *and is absorbed* (39–41).

It should be clearly understood that irradiation and photon dose “delivery” functions as a “trigger” such that the free-radical addition reaction continues *after* the light has been switched OFF. However, the reaction continues *only in regions of the material initially reached by photons* and thus where free radicals have been generated. The initial phase of the reaction kinetics is marked by an auto-acceleration until a point is quickly reached when auto-deceleration sets in and further progress occurs increasingly slowly (42, 43). By this point the material is transitioning into the glassy state, and internal movement of residual free radicals is slow (3, 4).

Once the composite has reached a hard glassy consistency, slow continued polymerization of the resin-phase is manifested by an increase in surface and bulk properties. Thus, surface hardness is known to gradually increase over periods of 1 month, or longer. However, intra-orally, water sorption may serve to soften surface layers (44).

### 6.3. “Bleaching” of photoinitiators and colour stability of BF-RBCs

Widely used type II photoinitiators, such as camphorquinone (CQ), are yellowish compounds precisely because they absorb blue wavelengths from white light. When CQ molecules react photochemically they are “destroyed” and so lose their yellow appearance. This is termed “bleaching” of the PI. Ideally the amount of CQ formulated is just sufficient for the photochemical reaction, leaving no residual CQ. Otherwise, the RBC may have an undesired yellow appearance. Additionally, CQ is used with an amine molecule (co-initiator). Again, residual amines can change chemically and develop a yellow appearance over time, thereby affecting the colour stability of the RBC. Managing this situation by the clinician is mainly down to: (a) being aware of the potential problem and (b) selecting RBC products that are known to be less susceptible to this problem.

### 6.4. Advanced requirements for photoinitiators and RBC formulations

Although we have suggested that many aspects of dental photo-curing can be straightforwardly explained, there are other aspects that are highly complex. A particularly detailed review of this situation is provided by Palin et al. (45). During the polymerization process, the refractive index (n) of the resin phase changes, as does the refractive index mismatch (Δ*n*) between resin and filler phases, which affects light-scattering and thus irradiance. Moreover, the composite densifies (see 7.1). As the absorbing species are consumed by the photocuring process there is a complex dynamic effect on the change in transported light, which results in a polymerization “wavefront”. Surprisingly, light transmittance can increase with prolonged irradiation times in some RBCs (45).

As regards clinical design requirements, it might be thought that the uppermost layers of the restoration should be, or become, semi-translucent to readily achieve greater light penetration and monomer conversion at depth. However, it is aesthetically disadvantageous to create a translucent restoration that fails to match the optical properties of the surrounding tooth.

Given the possibilities of using different photoinitiators, with differing molar absorptivities (extinction coefficients) and responding to different wavelengths from “multi-chip” LEDs, the design possibilities for controlling light penetration and the polymerization “wavefront” are considerable. It is not feasible to summarize this complex situation in a set of simple generalizations.

## 7. Degree of conversion

The kinetics (speed) of the polymerization process can be followed in a science lab by several complementary techniques. These include infra-red spectroscopy and monitoring shrinkage changes of the RBC that generally keep in step with the underlying polymerization reactions.

**Figure 9:**
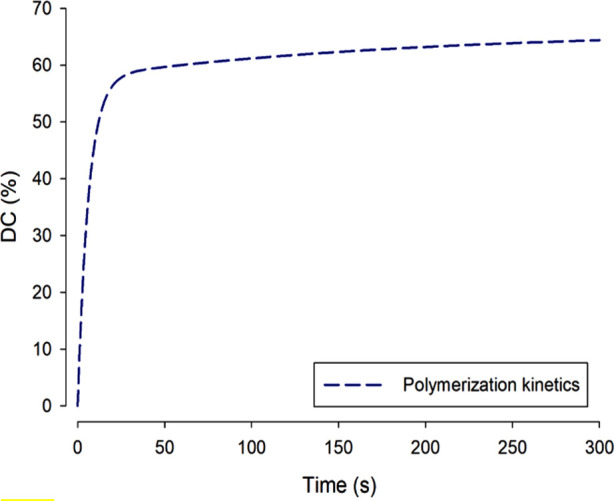
During dimethacrylate photopolymerization, as shown over a linear timescale, the *degree of conversion* normal increases rapidly and then—more slowly—approaches a maximum value: *ca*. 60%. Complete 100% conversion is not reached at oral temperatures because formation of the cross-linked network is increasingly a self-limiting process as the material converts from a mobile paste to a hard solid.

The most widely referenced quantity for expressing the immediate molecular “success” of photopolymerization is the Degree of Conversion (DC). The DC of a composite surface or thin film is the percentage of C = C *double*-bonds within the monomer molecules that have “disappeared” or rather converted to C-C single-bonds by polymerization. DC is measurable by infra-red, Raman and NMR spectroscopy (46, 47). For a well-polymerized dimethacrylate composite, DC is typically in the range of 60%–70%, not at all close to 100% (Figure 9). However, this type of DC data is not absolute, and differing chemistries and methods employed (in terms of spectral peaks used to monitor DC) vary widely between operators.

The reason for DC % values being much less than 100% is that polymerization of these crosslinking molecules is a *self-limiting* process. As the monomer begins to polymerize, viscosity rises rapidly and within seconds the material has vitrified (entered the glassy state of matter), so the network becomes topologically entangled and the chain-segmental mobility that is requisite for further reaction is either greatly reduced or becomes impossible.

DC is the main parameter conventionally used to express the state of the polymer network in RBCs. However, even starting with the same monomers *different network structures* may be generated that nevertheless have the *same* DC. This will be the case if the different structures exhibit variations in their cross-link densities. Such an outcome can arise by using ultra-rapid curing vs. slower photo-curing. Solvent swelling measurements can give an indication of such differences. More exact characterization involves x-ray diffraction experiments using synchrotron light sources.

### 7.1. Shrinkage phenomena

During polymerization of dimethacrylate monomers, the conversion of C = C bonds produces an intrinsic densification or shrinkage as the original *inter*-molecular spacings between individual monomer molecules are replaced by shorter C-C bonds creating the polymeric network chains. When the proportion of the resin-monomer phase is reduced, by addition of filler particles, the overall shrinkage is reduced. Nevertheless, even the most optimal RBC formulations exhibit some shrinkage. Shrinkage by itself is not the problem, but shrinkage stress—that arises when the RBC is photo-cured in the confined space of a cavity with adhesive bonding. When non-bulk fill composites are placed in a deep cavity, the traditional means of mitigating stress is to place the material incrementally. Bulk fill composites are intended to obviate the necessity for incremental placement. The good news is that, with many recent formulations, shrinkage phenomena are moderate (48). Manufacturers have striven to design and formulate against excessive shrinkage. Since RBC placement is both an art and a science the practitioner can resolve to learn more about optimal placement with different cavity shapes, sizes and designs.

### 7.2. Photo-curing of highly filled systems following pre-heating or sonication

There are several highly filled composite systems available that require either pre-heating (49) or sonication before bulk placement. The effect of these pre-treatments is to enhance flowability and thus reduce the viscosity to ensure good cavity adaptation. Once placed in the cavity these materials revert to a stiff and carveable consistency. When the desired occlusal anatomy has been achieved it is vital to proceed to apply the recommended photo-cure procedures. Without that essential step, clinical failure is certain because the composite paste would remain uncured!

### 7.3. Depth of Cure

When a clinician photo-cures a resin-composite in an occlusal cavity, a hard occlusal surface of the restoration is expected. However, if insufficient light has penetrated then the lower portion of the restoration may be uncured and soft. Bulk fill composites are, by definition, those having a Depth of Cure (DoC) of 4 mm or greater. The practitioner should note specific manufacturer claims for each product. These should include the precise irradiation regime that should be followed. Depth of Cure can be verified and validated either using precision laboratory instrumentation or roughly estimated in the dental clinic using simpler equipment and methods but with greatly reduced quality assurance.

A suitable plastic or metal mould may be used. For example, a circular hole of *ca.* 5 mm diameter may be drilled into a plastic sheet of either 2 mm or 4 mm thickness. The 4 mm depth may be used for a bulk fill resin composite. The hole (“cavity”) may be filled with the composite paste and flat surfaces produced above and below, using matrix strip and glass microscope slides. Following irradiation from the upper or “occlusal” surface, the hardness can be measured in a materials science lab on both the top and bottom surfaces, to give the values: *H_T_* and *H_B_*. These can then be re-expressed to give the *relative bottom hardness*: {*H_B_*/*H_T_*}.100% Ideally this ratio should be >95%. Uncured regions of the specimen can be made apparent by dissolving them with a solvent such as chloroform. More precise measurements can be made *via* hardness /depth profiles using slotted moulds (50).

The relative top/bottom hardness may be very roughly estimated using the type of plastic mould mentioned above. A sharp probe should not make a significant indentation or scratch on the *lower* surface of the composite. The experiment can be conducted immediately post-irradiation or, more realistically, after 24 h, allowing sufficient time for hardness development.

### 7.4. Heat generation by LCU and polymerization exotherm

Photons that are neither reflected, absorbed or scattered are thus transmitted. Their energy can finally be transformed into thermal heat, producing localised temperature rises (22–24). The power of some modern chips in dental LCUs is so high that they can burn mucosa! (25) Moreover, the polymerization process is itself exothermic.

When heat is generated within a material substance, *via* incident radiant energy and/or exothermic processes, two “extreme” situations may be distinguished—as regards any temperature change (**ΔT**). These are, firstly, an *isothermal* process, where the temperature of the system remains constant, so **ΔT **= **0**. For this to occur, the heat transfer must be slow enough for heat to be transferred completely to the surroundings. This might possibly arise in dentistry through application of a cold-water jet. The opposite extreme is an *adiabatic* process, where *the system exchanges no heat* with the surroundings and therefore the system *temperature will increase* as a function of time, during the finite period of heat generation: **ΔT(t) ≠ 0**. During dental photopolymerization of RBCs, the isothermal situation should not arise. If water-cooling were attempted this would be detrimental to effective polymerization as moderate **ΔT** increase is desirable to promote autoacceleration and attainment of solid vitrification.

## 8. The reciprocity hypothesis

As noted in Equation 3, above, the light energy applied to the material is, by definition, the product of **irradiance (I)** and irradiation **time (t)**. The first photo-cured dental resin-composites were considered to require irradiation for **t **= 60 s. Subsequent developments have enabled irradiation times to be reduced from 60 > 40 > 20 > 10 s, or—with specially formulated RBCs—even shorter times: >5 > 3 s.

To some extent, there has been an implicit assumption of a *general reciprocity hypothesis* that: “the same photo-cure outcomes will result from applying essentially constant energy densities despite reciprocal variations in the irradiance and time-period” (51–56). An assumption is thereby made that *if the irradiance is increased sufficiently* the irradiation period may be *reduced proportionately* without incurring inadequate consequences. In general, this reciprocity assumption is over-optimistic and could be seriously misleading. Resin-composites are not all created equal. Some have been specially formulated with advanced photoinitiator systems to permit ultra-rapid cure within 5 or even 3 s (54, 57). In other cases, it has been proposed that there is theoretical and experimental support for reciprocity to apply to monomer systems incorporating Type I photoinitiators (52) or to composites possessing a certain range of viscosity (55). But in other cases, there may be anomalies (53) or reciprocity only to a limited extent (56).

On this point some conclusions may be drawn:

• For some specially formulated RBCs and with matched LCUs, ultra-rapid cure may be safe and feasible.
• For most composites on the market, it is best not to assume exact reciprocity but to apply a safety factor of at least 2, especially with darker composite shades. That means irradiating for *at least double* the time corresponding to exact reciprocity. And even then, a means of checking the radiant emittance of the LCU is essential.

## 9. How the clinician can make light-curing work

It is essential to perform careful light-curing procedures since the consequences of failure are serious (Table 1). In particular, inadequate curing at depth into the material creates the potential for significant monomer elution (58, 59).

**Table 1:** Adverse consequences of under-cured resin composites.

• Breakdown of the resin matrix. • Increased wear & fracture. • Increased elution of monomer, etc. • Colour changes. • Reduced bond strengths. • Increased bacterial colonization. • Secondary caries.

When it is feasible to obtain a new light-curing unit, there are several considerations relating to the *selection criteria*.

*Firstly*, the LCU should be purchased from a reputable company that can supply detailed design and performance information upon request. Details of available operating modes: radiant emittance levels and irradiation times should be clear with some indication on the device itself. Beam profile information should be requested and may also be available from specialist companies, for example: BlueLight Analytics Inc (Halifax, NS, Canada). If the profile conforms to a “top hat” pattern (Figure 3) that is beneficial, other aspects being equal. However, there are many well-designed and high-quality devices that have not incorporated optics to reduce the inhomogeneity of the light output.

*Secondly*, the output wavelength spectrum, in the range 400–550 nm, should be requested. This will either be single-peak or multi-peak and thus inform regarding the corresponding photoinitiator absorption bands (Figure 7).

*Thirdly*, the overall design type should be noted. Some types have an extended optic tip (Figure 4); others emit light perpendicular to the long axis of the device (Figure 6). These can make for different levels of angular accessibility, particularly to posterior teeth. Both cable-free and cabled designs of LCU are available.

*Fourthly*, the divergence of the beam can be assessed by incidence onto a pad of white paper for several tip-to-target distances in the range of 0–10 mm. Related to this is the question of the beam area in relation to the area of an occlusal restoration. Note whether single or multiple positions are necessary for restoration coverage. Each clinic should have a portable radiometer for checking irradiance. Some designs are available that allow for different optic-tip areas.

In some cases, a clinician simply must work with the LCU provided to them. However, they can inspect the optic tip for freedom from damage and/or adhered composite material. Moreover, several of the checks mentioned above may still be conducted.

It is also possible to assess the performance of the LCU in achieving “Bulk Fill” (minimum 4 mm) Depth of Cure with the selected resin-composite(s), as mentioned above (section 7.3).

Even with a top-quality LED-LCU, careless usage may fail to deliver the expected irradiance levels throughout the material depth and/or across the restoration surface area. Clinical educationalists should consider the benefits of a device such as the MARC™-Patient Simulator, developed by Professor Richard Price and available from BlueLight Analytics (Halifax, NS, Canada). This can greatly facilitate the psycho-motor training of clinical staff undertaking intra-oral photo-curing procedures (41).

In this connection the issues of shortening the irradiation period by use of high-power LED-LCUs are significant, as discussed above (section 8). It is arguably easier to maintain the requisite concentration for shorter irradiation periods. Adequate eye protection must be used to facilitate the same levels of attention, as is also essential when using a dental handpiece for cavity preparation. The clinician should also avoid using high-power LCUs for long irradiation periods to protect the pulp from thermal damage.

Light curing in dentistry is now a mature technology. Nevertheless, further innovations can be anticipated. And, as with a powerful motor car, such devices require competent and highly skilled drivers!
